# Structural basis of the interaction between cyclodipeptide synthases and aminoacylated tRNA substrates

**DOI:** 10.1261/rna.075184.120

**Published:** 2020-11

**Authors:** Gabrielle Bourgeois, Jérôme Seguin, Morgan Babin, Muriel Gondry, Yves Mechulam, Emmanuelle Schmitt

**Affiliations:** 1 Laboratoire de Biologie Structurale de la Cellule, BIOC, Ecole polytechnique, CNRS, Institut Polytechnique de Paris, 91128 Palaiseau cedex, France; 2 Université Paris-Saclay, CEA, CNRS, Institute for Integrative Biology of the Cell (I2BC), 91198, Gif-sur-Yvette, France

**Keywords:** transfer RNA, Rossmann fold, aminoacyl-tRNA synthetases, cyclodipeptides, nonribosomal peptide synthesis

## Abstract

Cyclodipeptide synthases (CDPSs) catalyze the synthesis of various cyclodipeptides by using two aminoacyl-tRNA (aa-tRNA) substrates in a sequential mechanism. Here, we studied binding of phenylalanyl-tRNA^Phe^ to the CDPS from *Candidatus Glomeribacter gigasporarum* (*Cglo*-CDPS) by gel filtration and electrophoretic mobility shift assay. We determined the crystal structure of the *Cglo*-CDPS:Phe-tRNA^Phe^ complex to 5 Å resolution and further studied it in solution using small-angle X-ray scattering (SAXS). The data show that the major groove of the acceptor stem of the aa-tRNA interacts with the enzyme through the basic β2 and β7 strands of CDPSs belonging to the XYP subfamily. A bending of the CCA extremity enables the amino acid moiety to be positioned in the P1 pocket while the terminal A76 adenosine occupies the P2 pocket. Such a positioning indicates that the present structure illustrates the binding of the first aa-tRNA. In cells, CDPSs and the elongation factor EF-Tu share aminoacylated tRNAs as substrates. The present study shows that CDPSs and EF-Tu interact with opposite sides of tRNA. This may explain how CDPSs hijack aa-tRNAs from canonical ribosomal protein synthesis.

## INTRODUCTION

**FIGURE 1.**
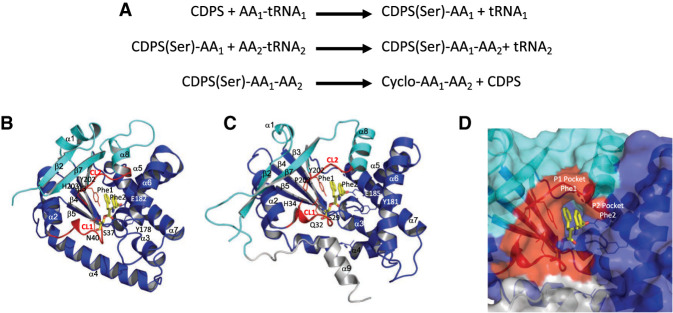
Catalytic cycle and binding of the dipeptide to CDPS. (*A*) Catalytic cycle of CDPS. The aminoacyl-enzyme intermediate is bound to a serine residue. (*B*) Structure of *Snou*-CDPS (formerly called AlbC) bound to a Phe–Phe dipeptide. The model is deduced from the structure of *Snou*-CDPS bound to a reaction intermediate (PDB ID code 4Q24) (Moutiez et al. 2014a; Schmitt et al. 2018). The first part of the Rossmann fold is colored in cyan and the second part is in dark blue. The two catalytic loops CL1 and CL2 are in red. Residues important for catalysis are shown in sticks. The dipeptide bound to the catalytic serine is in yellow stick. *Snou*-CDPS is a member of the NYH-CDPS subfamily (Jacques et al. 2015; Bourgeois et al. 2018). (*C*) Structure of *Rgry*-CDPS bound to a Phe–Phe dipeptide. The same color code as in view *B* is used. *Rgry*-CDPS is a member of the XYP-CDPS subfamily. (*D*) Closeup of the *Rgry*-CDPS dipeptide binding site from view *C*. The molecular surface is represented. The view shows the two substrate binding pockets named P1 and P2 (see text).

Cyclodipeptide synthases (CDPSs) are enzymes that use sequentially two aminoacyl-tRNAs (aa-tRNAs) to catalyze the formation of two peptide bonds leading to the production of various cyclodipeptides (Fig. 1A; Gondry et al. 2009; Canu et al. 2019). Cyclodipeptides can then be modified by cyclodipeptide-tailoring enzymes in biosynthetic pathways responsible for the production of diketopiperazines with interesting biological activities (Gondry et al. 2009; Aravind et al. 2010; Belin et al. 2012; Borthwick 2012; Giessen and Marahiel 2014; Borgman et al. 2019).

CDPSs are built around a Rossmann fold similar to the catalytic domain of the two class Ic aminoacyl-tRNA synthetases (aaRSs), TyrRS and TrpRS. The catalytic mechanism of CDPSs was extensively investigated (Vetting et al. 2010; Bonnefond et al. 2011; Sauguet et al. 2011; Moutiez et al. 2014a). In particular, the structure of the CDPS from *Streptomyces noursei* (*Snou*-CDPS, formerly called AlbC) complexed with a reaction intermediate was determined (Fig. 1B–D; Moutiez et al. 2014a). Overall, the data show that the first aa-tRNA binds the enzyme with its aminoacyl moiety accommodated in a P1 pocket. The aminoacyl moiety is transferred onto a conserved serine residue (S37 in *Snou*-CDPS) to form an aminoacyl-enzyme intermediate (Sauguet et al. 2011). The second aa-tRNA interacts with the intermediate and its aminoacyl moiety, accommodated in a wide P2 pocket, is transferred to the aminoacyl-enzyme to form a dipeptidyl-enzyme intermediate (Fig. 1D). Finally, the dipeptidyl moiety undergoes an intramolecular cyclization involving a tyrosine (Y202 in *Snou*-CDPS), properly positioned to favor the nucleophilic attack at the enzyme ester bond, leading to the cyclodipeptide (Moutiez et al. 2014a; Schmitt et al. 2018). In addition to S37 and Y202, residues Y178 and E182 (*Snou*-CDPS numbering) participate in the catalysis. Y178 and E182 are involved in anchoring the amino and carboxyl groups of the first substrate during the catalytic cycle. Moreover, E182 would act as a catalytic base essential for dipeptidyl-enzyme formation. Notably, the catalytic residues S37, Y178, E182, and Y202 are almost strictly conserved in all active CDPSs (Fig 1B,C; Jacques et al. 2015).

CDPSs are divided into two phylogenetically distinct subfamilies named NYH-CDPSs and XYP-CDPSs according to the occurrence of two sets of conserved residues (Fig. 1B,C; Jacques et al. 2015). Determination of crystal structures of four NYH-CDPSs and three XYP-CDPSs made it possible to give structural bases for the partition of the CDPSs into the two subfamilies (Bourgeois et al. 2018). XYP-CDPSs and NYH-CDPSs mainly differ in the first half of their Rossmann fold. However, the catalytic residues adopt similar positioning regardless of the subfamily (Fig. 1B,C; Bourgeois et al. 2018). Despite these data, interaction between CDPSs and their two aa-tRNA substrates remains poorly documented. Previous work on the NYH *Snou*-CDPS showed that the enzyme discriminates between the two aa-tRNA substrates and possess a specific binding site for each. The binding of the first substrate is strongly dependent on the identity of the aminoacyl moiety of the aa-tRNA whereas both the aminoacyl moiety and the tRNA sequence are essential for the specific recognition of the second substrate (Moutiez et al. 2014b). Nevertheless, no structural data describing a complex between a CDPS and an aa-tRNA was available.

Here, we study the binding of *E. coli* phenylalanyl-tRNA^Phe^ (Phe-tRNA^Phe^) to the XYP-CDPS from *Candidatus Glomeribacter gigasporarum* (*Cglo*-CDPS) by gel filtration and electrophoretic mobility shift assay. We determine the crystal structure of the *Cglo*-CDPS:Phe-tRNA^Phe^ complex to 5 Å resolution and further validate it by solution studies using small-angle X-ray scattering (SAXS). Despite the modest resolution, structural homologies with other enzymes from the XYP subfamily known at high-resolution allowed us to deduce more informative models for the binding of the aa-tRNA. The data show that the aa-tRNA interacts with the enzyme through the basic β2 and β7 strands of the XYP-CDPS subfamily. A bending of the CCA extremity enables the aminoacyl moiety to be positioned in the P1 pocket while the terminal A76 adenosine occupies the P2 pocket. In cells, CDPSs and the elongation factor EF-Tu (also called EF1A in bacteria, IUBMB 1996) share aminoacylated tRNAs substrates. The present study shows that CDPSs and EF-Tu interact with opposite sides of tRNA. This may explain how CDPSs hijack aa-tRNAs from canonical ribosomal protein synthesis.

## RESULTS

### Characterization of *Cglo*-CDPS-S32A:Phe-tRNA^Phe^ complex

**FIGURE 2.**
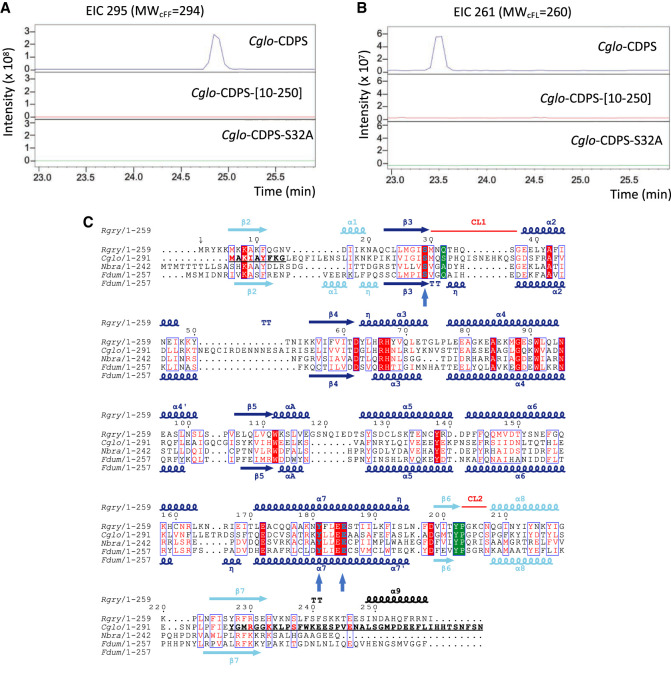
*Cglo*-CDPS. (*A*,*B*) Determination of the cyclodipeptide-synthesizing activities of *Cglo*-CDPS by LC-MS analyses. Extracted ion chromatograms (EIC) corresponding to the m/z of cFF (*A*) and cFL (*B*) from the supernatants of *E. coli* cells expressing wild-type *Cglo*-CDPS (blue) and its variants *Cglo*-CDPS-[10-250] (red) and *Cglo*-CDPS-S32A (green) are displayed. (*C*) Sequence alignment of *Cglo*-CDPS with three other XYP-CDPSs of known 3D structure. The figure was drawn with Espript (Gouet et al. 1999). Secondary structures of *Rgry*-CDPS are indicated at the *top* of the alignment and those of *Fdum*-CDPS are indicated at the *bottom*. The first half of the Rossmann fold is colored in cyan and the second half of the Rossmann fold is colored in blue. Residue characteristics of the XYP-subfamily are in green boxes. Other important residues (S29, E185, Y181, *Rgry*- numbering) are shown in bold blue and indicated by a *vertical* arrow. Residues displayed in bold in β2 and β7 of *Cglo*-CDPS sequence indicate the deleted region in the *Cglo*-CDPS-[10-250] variant.

We used *Cglo*-CDPS to study aa-tRNA binding. The ability of *Cglo*-CDPS to produce cyclo(l-Phe-l-Phe) (cFF) and cyclo(l-Phe-l-Leu) (cFL) in *E. coli* was first measured in Jacques et al. (2015). Here, we confirmed the enzymatic activity of *Cglo*-CDPS in *E. coli*. Moreover, we showed that the replacement of the conserved catalytic serine residue S32 by an alanine (*Cglo*-CDPS-S32A) inactivated the enzyme (Fig. 2A,B). As previously demonstrated for many CDPSs, this mutation impairs the formation of the aminoacyl-enzyme at the first step of catalysis (Vetting et al. 2010; Bonnefond et al. 2011; Sauguet et al. 2011; Moutiez et al. 2014a; Bourgeois et al. 2018). Therefore, this variant allows formation of a CDPS:aa-tRNA complex without further reaction.

**FIGURE 3.**
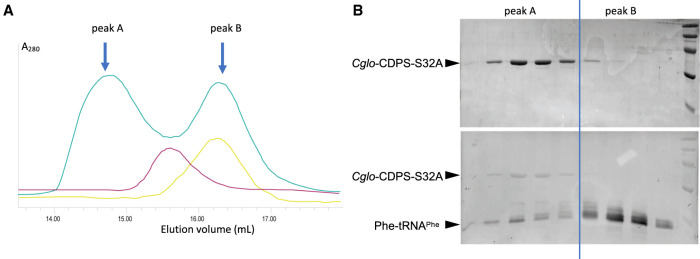
Gel filtration analysis of *Cglo-*CDPS:aa-tRNA mixtures. (*A*) Gel filtration chromatograms for *Cglo-*CDPS-S32A (pink), Phe-tRNA^Phe^ (yellow), *Cglo-*CDPS-S32A:Phe-tRNA^Phe^ mixture (green). (*B*) SDS-PAGE analysis of fractions from peak A and peak B. Gels were stained using ethidium bromide (*lower* part) and then Coomassie blue (*upper* part). The molecular weight marker (GE-Healthcare) is shown to the *right* of the gel. Visible bands correspond to proteins with molecular weights of 97, 66, 45, 30, 20 kDa from *top* to *bottom*. Experimental conditions are described in the Materials and Methods section.

*E. coli* Phe-tRNA^Phe^ (Supplemental Fig. S1A) was aminoacylated using *E. coli* PheRS and then purified (Materials and Methods). In order to test for complex formation, 1.2 molar excess of aminoacylated or nonaminoacylated tRNA^Phe^ were mixed with *Cglo*-CDPS-S32A variant. After 15 min of incubation at 4°C, the complexes were analyzed by size exclusion chromatography (Fig. 3A; Supplemental Fig. S2A). Eluted fractions were analyzed by SDS-PAGE and gels were successively stained with ethidium bromide and Coomassie blue to reveal tRNA and *Cglo*-CDPS, respectively (Fig. 3B; Supplemental Fig. S2B). As shown in Figure 3A, the elution profile of *Cglo*-CDPS-S32A and Phe-tRNA^Phe^ mixture gave two peaks (peaks A and B). Peak A contained the *Cglo*-CDPS-S32A:Phe-tRNA^Phe^ complex whereas peak B contained the excess of Phe-tRNA^Phe^ (Fig. 3B). Interestingly, when nonaminoacylated tRNA^Phe^ was mixed with *Cglo*-CDPS-S32A, two less resolved peaks (peak C and D) were observed (Supplemental Fig. S2A). SDS-PAGE analysis showed that Peak C contained *Cglo*-CDPS-S32A and tRNA^Phe^ whereas Peak D contained tRNA^Phe^ with low amounts of *Cglo*-CDPS-S32A (Supplemental Fig. S2B). This suggested that a dynamic equilibrium between *Cglo*-CDPS-S32A and tRNA^Phe^ occurred during the chromatography and therefore that the complex formed with nonaminoacylated tRNA^Phe^ was less stable than that formed with Phe-tRNA^Phe^.

**FIGURE 4.**
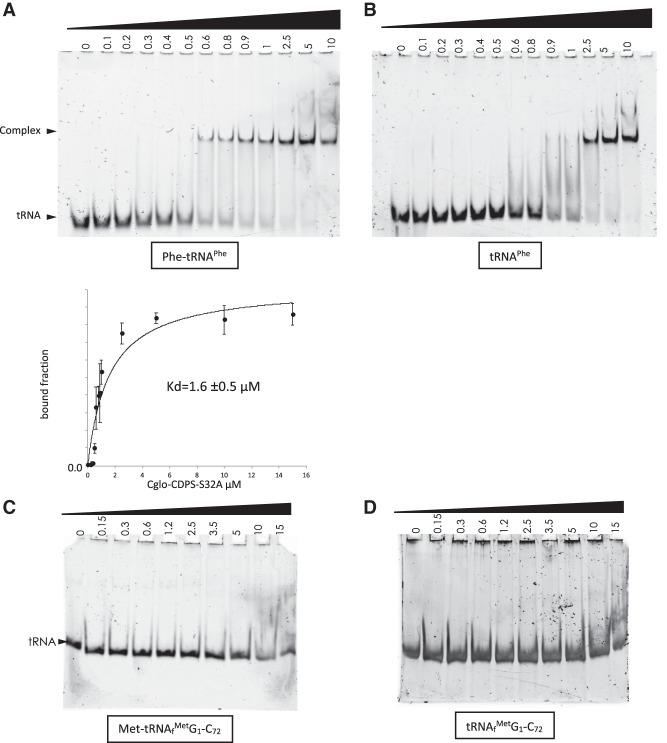
Electrophoretic mobility shift assays of *Cglo*-CDPS-S32A:tRNA mixtures. (*A*) *Cglo*-CDPS-S32A was incubated at increasing concentrations (0.1–15 µM) with 75 nM Phe-tRNA^Phe^ and electrophoresed on a native acrylamide gel. The panels show typical experiments. All gels were stained using SYBR gold (Invitrogen) and then Coomassie blue. The curve *below* the gel corresponds to a plot of the intensity of the bound fraction as a function of enzyme concentration. It was fitted according to a simple binding equilibrium. A *K*_D_ value of 1.6 ± 0.5 µM was deduced from three independent experiments. (*B*) Same experiment as that in *A*, with 75 nM of tRNA^Phe^. No *K*_D_ value was derived because tRNA dissociation from CDPS is visible during migration. (*C*) Same experiment as that in *A*, with 75 nM Met-tRNA^Met^. (*D*) Same experiment as that in *A*, with 75 nM tRNA^Met^.

We then used Electrophoretic Mobility Shift Assay (EMSA) to evaluate tRNA binding affinity to the enzyme. Using *Cglo*-CDPS-S32A and Phe-tRNA^Phe^, we observed the gradual appearance of a shifted band when increasing enzyme concentrations were used (Fig. 4A). As revealed by SYBR gold (Invitrogen) and Coomassie blue staining, this band contained both *Cglo-*CDPS-S32A and Phe-tRNA^Phe^. An apparent *K*_D_ value of 1.6 ± 0.5 µM was deduced from three independent titration experiments. When nonaminoacylated tRNA^Phe^ was used significant dissociation of the CDPS:RNA complex occurs during electrophoresis (Fig. 4B). Therefore an accurate *K*_D_ value could not be derived. However, comparison of the EMSA gels (Fig. 4A,B) indicated that apparent tRNA binding affinity for *Cglo*-CDPS-S32A was smaller for the nonaminoacylated form, in agreement with the size-exclusion chromatography experiments. This result pointed out the importance of the phenylalanyl moiety of Phe-tRNA^Phe^ for binding to *Cglo*-CDPS-S32A. Finally, to further investigate if the method allowed us to evidence specific binding of Phe-tRNA^Phe^ to *Cglo*-CDPS-S32A, we used Met-tRNA_f_^Met^G_1_-C_72_ and nonaminoacylated tRNA_f_^Met^G_1_-C_72_ (Fig. 4C,D; Supplemental Fig. S1B). This tRNA was used because it is highly overproduced in *E. coli* and easy to purify. It contains a G_1_–C_72_ base pair instead of the A_1_–U_72_ original one to make it an elongator tRNAs (Guillon et al. 1993). As shown in Figure 4C,D, no shifted tRNA band was visible even at high enzyme concentration. Overall the results indicate that *Cglo*-CDPS-S32A specifically binds Phe-tRNA^Phe^ with a *K*_D_ value in the micromolar range.

### Structure of overproduced *E. coli* tRNA^Phe^

*Cglo*-CDPS-S32A:Phe-tRNA^Phe^ complex purified by gel filtration (peak A in Fig. 3A) was used for crystallization using standard sitting drop screening (Hampton research). However, during the search for crystals containing *Cglo*-CDPS-S32A:Phe-tRNA^Phe^ complex, we identified crystals containing only tRNA^Phe^ (Table 1). In a first step, we used these crystals to determine the structure of tRNA^Phe^ overproduced in *E. coli* at 3.1 Å resolution (Table 1, Materials and Methods). The structure of the overproduced *E. coli* tRNA^Phe^ contains nucleotides 1 to 75 (Supplemental Fig. S3). Among the 10 modified nucleosides of mature *E. coli* tRNA^Phe^ (Juhling et al. 2009), 3-(3-amino-3-carboxypropyl) uridine (acp^3^U) was visible at position 47 (Supplemental Fig. S3B). 4-thiouridine (S^4^U) at position 8, dihydrouridine (D) at positions 16 and 20, pseudouridine (ψ) at positions 32, 39, and 55 were also modeled because these modifications are isosteric with the original bases although, at 3.1 Å resolution, no further validation was available in the electron density map. Ribothymidine 54 present in most tRNAs was also modeled. Finally, 2-methylthio-*N*^6^-isopentenyladenosine (ms^2^i^6^A) at position 37, 7-methylguanosine (m^7^G) at position 46 were not modeled because the modifications were not visible in the electron density map. Comparison of the tRNA^Phe^ structure with that of unmodified tRNA^Phe^ (Byrne et al. 2010) shows that the two molecules are very close to one another (rmsd = 2.56 Å for 1572 atoms compared). In particular, both tRNAs harbor a G10–C25–G44 triplet base pair. Moreover, A26 is unpaired. U45 is flipped out of the tRNA core and forms Watson–Crick interactions with A26 of a symmetry-related molecule (Supplemental Fig. S3C). This packing interaction is notable since the present structure and that of unmodified tRNA^Phe^ were determined from different crystalline forms. Hence, in tRNA^Phe^, U45 may have a marked propensity to adopt the flipped-out conformation.

**TABLE 1.**
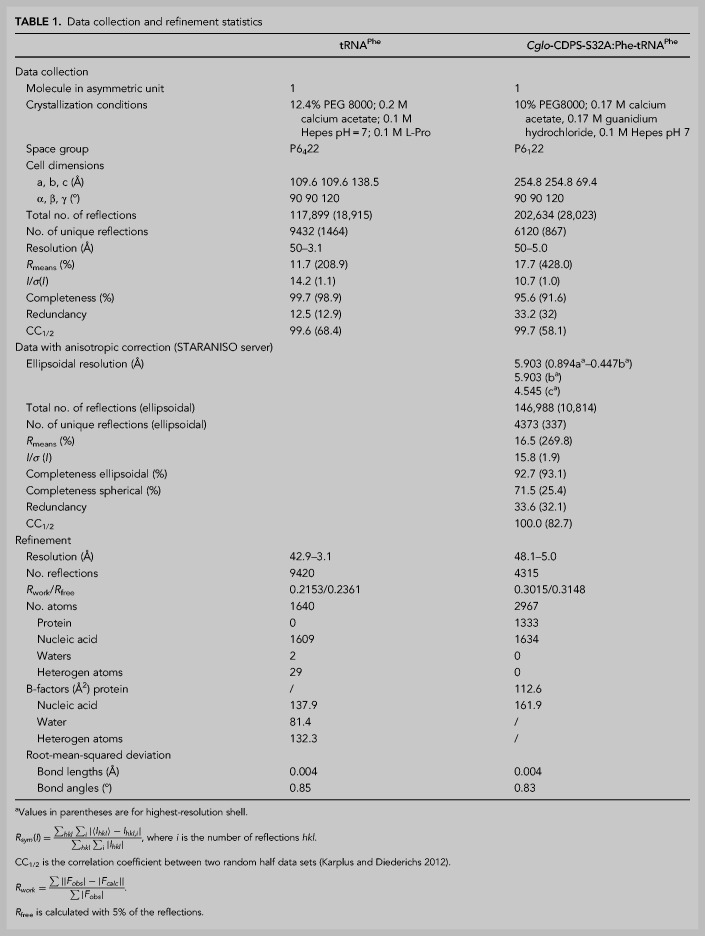
Data collection and refinement statistics

### Crystal structure of *Cglo*-CDPS-S32A:Phe-tRNA^Phe^ complex

A crystalline form diffracting to 5 Å resolution and containing the *Cglo*-CDPS-S32A:Phe-tRNA^Phe^ complex was obtained (Table 1). The structure was solved by molecular replacement using two search ensembles; one corresponding to the core domain of *Rgry*-CDPS (Bourgeois et al. 2018); residues 12 to 223, 28% identity with *Cglo*-CDPS, Fig. 2D) and the other one to residues 2 to 71 of the structure of *E. coli* tRNA^Phe^ described above. The quality of the molecular replacement density map was sufficient to adjust the models of the tRNA and of the backbone of the protein. Bases 72–75 of the tRNA were then positioned. To avoid overfitting, the protein was refined as a polyalanine model with only a few side chains modeled for residues visible in the electron density. In the final steps of the refinement procedure, weak residual density was visible in the active site at the position expected for the phenylalanyl moiety (Moutiez et al. 2014a). We therefore decided to construct a tentative model of the phenylalanylated A76 base using the structures of the *Snou*-CDPS (formerly called AlbC) bound to the analog mimicking the putative dipeptidyl intermediate, ZPCK (N-carbobenzyloxy-l-Phe-chloromethylketone, Moutiez et al. 2014a) and that of a Phe-tRNA^Phe^ molecule coming from a Phe-tRNA^Phe^:EF-Tu complex (Nissen et al. 1995) as guides. The final model was refined in Phenix (Afonine et al. 2012) to 5 Å resolution (Table 1).

### Architecture of the *Cglo-*CDPS-S32A:Phe-tRNA^Phe^ complex

**FIGURE 5.**
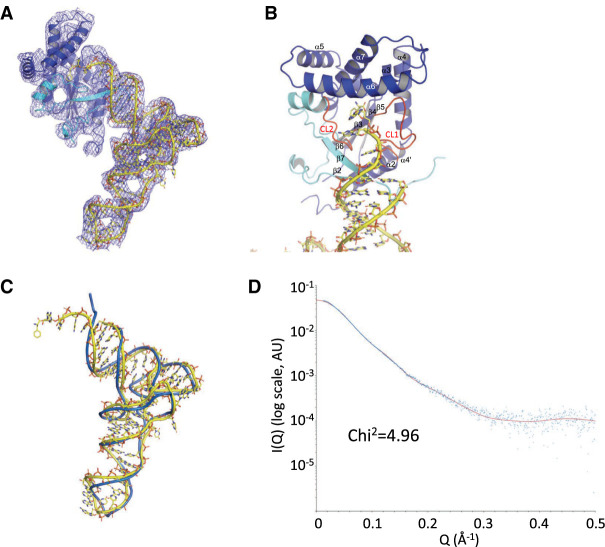
Structure of the *Cglo*-CDPS:Phe-tRNA^Phe^ complex. (*A*) *Cglo*-CDPS-S32A:Phe-tRNA^Phe^ complex. The first half of the Rossmann fold is colored in cyan and the second half is in dark blue. The 2mF_o_ − DF_c_ electron density map is contoured at 1 sigma. (*B*) Closeup of the catalytic center. The two catalytic loops CL1 and CL2 (see Fig. 1) are colored in red. (*C*) Superimposition of free tRNA^Phe^ onto Phe-tRNA^Phe^ in the *Cglo*-CDPS-S32A:Phe-tRNA^Phe^ complex. (*D*) The experimental SAXS curve (blue) is compared with the theoretical diffusion curve deduced from the crystal structure of the *Cglo*-CDPS-S32A:Phe-tRNA^Phe^ complex (red). AU, arbitrary units. See also Supplemental Figure S6.

The 5.0 Å resolution structure of the *Cglo*-CDPS-S32A:Phe-tRNA^Phe^ complex shows a Phe-tRNA^Phe^ molecule bound to a monomer of *Cglo*-CDPS. Consistent with the previously determined CDPS structures, *Cglo*-CDPS comprises a Rossmann fold with the active site located at the switch point between the two halves of the domain. Moreover, as evidenced by sequence alignments and structure superimposition, the first half of the Rossmann fold is characteristic of enzymes from the XYP subfamily with residues important for catalysis similarly positioned (Figs. 1A,B, 2C; Bourgeois et al. 2018). As compared to other XYP-CDPS sequences, *Cglo*-CDPS has two insertions: one of six residues downstream from β3 [34–40] and one of 10 residues downstream from α2 (Fig. 2C). The structure shows that *Cglo*-CDPS approaches Phe-tRNA^Phe^ from the major groove of the acceptor stem via the β2 and β7 strands of the first half of the Rossmann fold (Fig. 5A,B). The CCA arm is bent and located in a channel delineated by the two catalytic loops CL1 and CL2 (Fig. 5B). This contributes to the positioning of the aminoacyl group in the catalytic pocket of the enzyme.

Comparison of the structure of free tRNA^Phe^ with that of Phe-tRNA^Phe^ bound to *Cglo*-CDPS (Fig. 5C; rmsd = 3.8 Å for 1611 atoms compared), highlights the bending of the aminoacylated CCA end of Phe-tRNA^Phe^ that accompanies the positioning of the aminoacylated terminal adenosine 76 in the catalytic pocket. As illustrated in Supplemental Figure S4, other parts of the tRNA do not interact with the enzyme but are involved in crystal packing. Indeed, stacking interactions between tRNA molecules involve G34 from the anticodon and the G19:C56 base pair at the elbow. Moreover, the backbone of the variable loop of tRNA (G44 to U47) contacts the minor groove at the top of the anticodon helix of a symmetry-related molecule. Interestingly, a Watson–Crick interaction between U45 of one molecule and A26 of another molecule stabilizes this interaction, as also observed in the crystals containing only tRNA^Phe^ (Supplemental Fig. S3). Concerning the protein, packing interactions involve the α6 helix on one side and the αA helix on the other side (Supplemental Fig. S4). It should be noted that the acceptor helix of the tRNA is not involved in packing interactions. This makes it unlikely that crystal packing has influenced the architecture of the complex.

**FIGURE 6.**
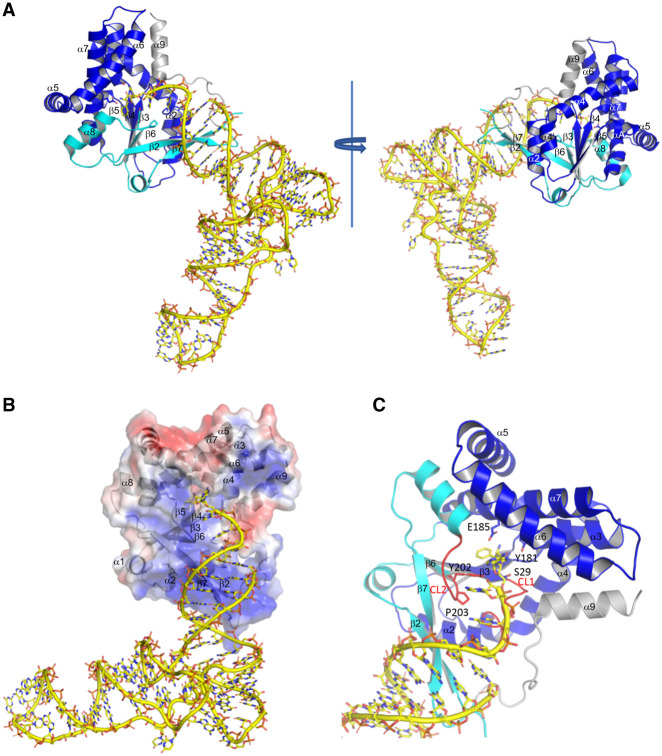
Model of the *Rgry*-CDPS:Phe-tRNA^Phe^ complex. (*A*) The structure of *Rgry*-CDPS refined at 2.0 Å resolution (PDB: 5MLP, Bourgeois et al. 2018) was superimposed onto that of *Cglo*-CDPS to deduce a model of *Rgry*-CDPS:Phe-tRNA^Phe^. The color code is the same as in Figure 5. The carboxy-terminal helix of *Rgry*-CDPS is in gray. The two structures are superimposed with an rmsd of 2.03 Å for 194 Ca atoms compared. (*B*) Electrostatic potential map of *Rgry*-CDPS (blue, positive; red, negative; white, neutral) with the tRNA shown in yellow sticks and cartoon. The views emphasize the binding of the acceptor arm of the tRNA by the positively charged protein area containing β2–β7 (see also Supplemental Fig. S4). The electrostatic potential map was calculated using the APBS plugin in Pymol (Schrodinger 2017). (*C*) Closeup of the catalytic center in the *Rgry*-CDPS:Phe-tRNA^Phe^ model. Figures 1, 5–7 were drawn with Pymol.

Because crystals diffracted to 5 Å resolution, the quality of the electron density did not allow us to build a full atomic model of *Cglo*-CDPS and some parts of the structure of the protein are missing in the model. Therefore, in order to better understand the interaction of tRNA with CDPS, we superimposed the structure of *Rgry*-CDPS (PDB: 5MLP, Bourgeois et al. 2018) onto that of *Cglo*-CDPS (rmsd of 2.03 Å for 194 Cα atoms compared). *Rgry*-CDPS is, like *Cglo*-CDPS, a member of the XYP subfamily that produces cFF and cFL (Jacques et al. 2015). Its structure is known at 2.0 Å resolution (Bourgeois et al. 2018). From the superimposition, we deduced a model of *Rgry*-CDPS bound to Phe-tRNA^Phe^ (Fig. 6). The model was further improved using geometry idealization procedures in phenix.dynamics (Afonine et al. 2012). This model shows that β2 and β7 strands are embedded into the major groove of the acceptor helix (Fig. 6B,C). The length and basic character of β2 and β7 strands are known to be features of the XYP-CDPS subfamily (Supplemental Fig. S5A; Bourgeois et al. 2018). These two strands contain basic stretches including a KϕXF consensus in β2 (where ϕ stands for hydrophobic residue) and a L(R/K)FKK consensus in β7 (Bourgeois et al. 2018). Consistent with these findings, the electrostatic potential of the protein highlights the basic character of the β2 and β7 strands that interact with the acceptor stem of the tRNA (Fig. 6B; Supplemental Fig. S5B). The positioning of the β2 and β7 strands may help to stabilize the bent conformation of the aminoacylated CCA end of Phe-tRNA^Phe^ (Figs. 5A,B, 6B,C).

In the *Rgry*-CDPS:Phe-tRNA^Phe^ model, the aminoacylated terminal A76 is located within the catalytic pocket. CDPSs possess two binding pockets, P1 and P2, accommodating the aminoacyl moieties of the first and second aa-tRNA, respectively (Moutiez et al. 2014a; Jacques et al. 2015; Bourgeois et al. 2018). Here, the phenylalanyl moiety occupies the P1 pocket near the residues known to be important for catalysis (S29,Y181,E185,Y202; Fig. 6C) while the adenosine moiety occupies the P2 pocket. The fact that the P2 pocket is wide and shallow likely explains why it is less specific for the amino acid and how it can accommodate the adenosine moiety of the first aa-tRNA substrate. Such a positioning of the aminoacylated part of the tRNA indicates that the present structure illustrates the binding of the first aa-tRNA to the enzyme.

### Solution studies using SAXS and site-directed mutagenesis

To further reinforce our data, and in particular to insure that the observed complex between *Cglo*-CDPS and tRNA was not the result of crystallization artifacts, the structure of *Cglo*-CDPS-S32A:Phe-tRNA^Phe^ was also studied in solution using SAXS coupled with size-exclusion chromatography (see Materials and Methods and Supplemental Fig. S6). Molecular mass of the complex in solution was estimated from the SAXS data to 53 ± 3 kDa using the consensus Bayesian approach (Hajizadeh et al. 2018) and to 58 kDa using the MoW method (Piiadov et al. 2019). This agrees with a 1:1 CDPS:tRNA complex (calculated molecular mass of 58 kDa), as observed by gel filtration (data not shown) and in the crystal structure. The experimental SAXS curve measured with the size-exclusion chromatography-purified complex was then compared with the curve calculated from the crystal structure coordinates (Fig. 5D). The overall agreement was good (X^2^ = 4.96) arguing in favor of the biological significance of our crystalline model. Small deviations between the two curves were however observed, in particular at low Q values where experimental diffusion is larger than expected. When not bound to tRNA, *Cglo*-CDPS tends to form aggregates that diffuse X rays at low Q (SAXS data not shown). Thus, one hypothesis to account for the small deviation of the two curves at low Q may be a very small amount of dissociation of the complex followed by interactions between the liberated protein molecules, in the path between the chromatography column and the SAXS cell.

To further validate the involvement of the β2 and β7 strands in the binding of Phe-tRNA^Phe^, we designed variants of *Cglo*-CDPS and *Cglo*-CDPS-S32A in which residues 1 to 9 and residues 251–293 were removed (see bold characters in β2 and β7 in *Cglo* sequence Fig. 2C). The variants were named *Cglo*-CDPS-[10-250] and *Cglo*-CDPS-S32A-[10-250]. First, cyclodipeptide-synthesizing activity was measured from overproducing cells as described in the Materials and Methods section. The presence of the truncated protein in the soluble fraction of crude extracts was confirmed by SDS-PAGE analysis (Supplemental Fig. S7A,B) but no cyclodipeptide-synthesizing activity was detected for the *Cglo*-CDPS-[10-250] variant (Fig. 2A,B). The truncated protein was purified and its correct folding was checked by circular dichroism (Supplemental Fig. S7C). In a second step, the ability of the *Cglo*-CDPS-S32A-[10-250] variant to bind Phe-tRNA^Phe^ was analyzed by gel filtration (Supplemental Fig. S7D). When aminoacylated tRNA^Phe^ and *Cglo*-CDPS-S32A-[10-250] variant were mixed using a 1.2:1 molar ratio, only one large peak with a maximum corresponding to the elution peak Phe-tRNA^Phe^ was observed, arguing in favor of the absence of complex formation (Supplemental Fig. S7D). This confirmed the importance of the β2 and β7 strands for tRNA binding, as indicated by the crystallographic structure.

## DISCUSSION

This work describes the first crystallographic structure of a *Cglo-*CDPS-S32A:Phe-tRNA^Phe^ complex at 5 Å resolution. The binding mode allows positioning of the esterified phenylalanine within the catalytic pocket close to the catalytic serine residue. Therefore, the structure illustrates the binding of the first aa-tRNA. Higher affinity of Phe-tRNA^Phe^ for the first site is at least partly explained by complementarity of the phenylalanyl moiety with P1. After reaction, Phe acylated to the catalytic serine will contribute to shape the otherwise wide P2 pocket thereby favoring binding of the second aa-tRNA.

The structure shows that the β2 and β7 strands of the first part of the Rossmann fold of *Cglo*-CDPS are involved in tRNA binding. Interestingly, this part of the CDPS structures has been previously shown to provide a structural basis for the partition of the CDPSs into the two XYP and NYH-CDPS subfamilies. The crystallographic structures of three other XYP-CDPSs are known. Among them, *Rgry*-CDPS catalyzes formation of cFF and cFL, like *Cglo*-CDPS. The experimental structure allowed us to readily derive a model for the binding of the tRNA to *Rgry*-CDPS, whose structure is known at high-resolution. This model shows how the β2 and β7 strands are embedded into the major groove of the acceptor stem of Phe-tRNA^Phe^. A docking model performed with all known XYP-CDPS structures highlights the role of the basic β-strands in tRNA selection for this subfamily (Fig. 6B,C; Supplemental Fig. S5B–D). This raises the question of tRNA recognition in the NYH-CDPS subfamily. Given the structural proximity of the two CDPS subfamilies, docking models of tRNA on the NYH-CDPS can also be deduced from the structure of *Cglo-*CDPS-S32A:Phe-tRNA^Phe^ complex (Supplemental Fig. S5E–G). The figure shows that the β2 and β7 strands, shorter in NYH-CDPS, do not provide a large and basic interface favorable to tRNA binding. Moreover, a previous study has proposed that the α4 helix of *Snou*-CDPS could be involved in tRNA binding (Moutiez et al. 2014b). Therefore, it cannot be excluded that the binding of the acceptor stem of the first aa-tRNA is different in the two CDPS subfamilies even if the aminoacylated A76 base would be accommodated in the same cavity. Further experiments and structural data are required to fully address this question.

**FIGURE 7.**
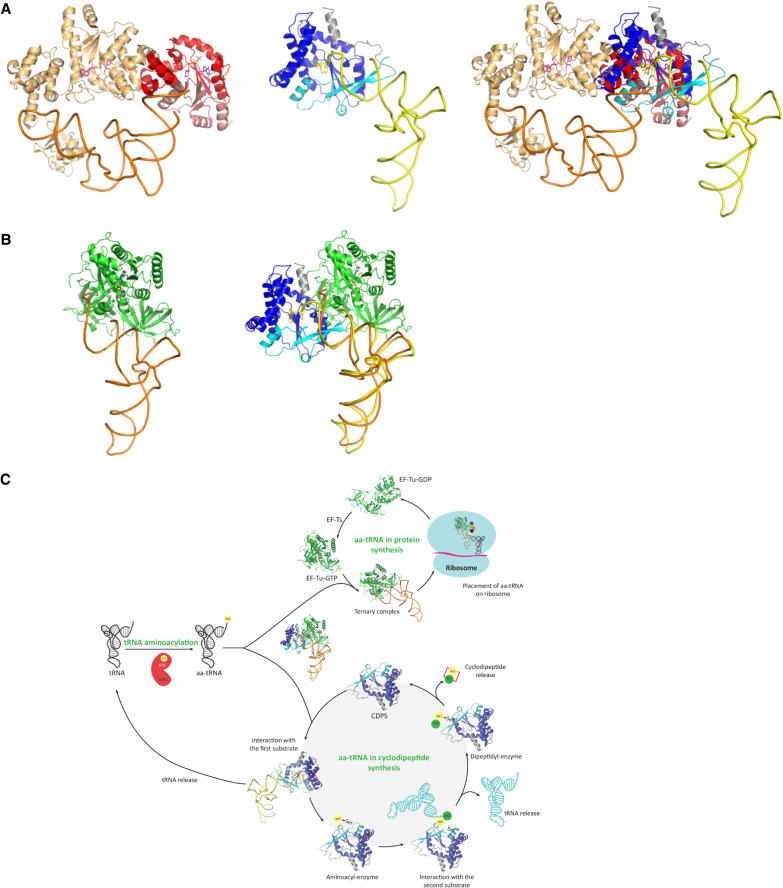
Comparison of tRNA binding modes. (*A*) Structural alignment of *Rgry*-CDPS:Phe-tRNA^Phe^ with *T. thermophilus* TyrRS:tRNA^Tyr^. *Left* view, the *T. thermophilus* TyrRS:tRNA^Tyr^ complex is represented in cartoon (PDB ID Code 1H3E, Yaremchuk et al. 2002). One monomer is in light orange and the amino-terminal domain of the second monomer is in colored in light red (first part of the Rossmann fold) and in dark red (second part of the Rossmann fold). ATP and tyrosine are shown in sticks. Middle view, *Rgry*-CDPS:Phe-tRNA^Phe^ model. Phe-A76 and C75 are shown in yellow sticks. The color code is the same as in Figure 5. *Right* view, superimposition of the two structures. The view shows that the tRNA binding mode is different for *Rgry*-CDPS and for TyrRS even if the two enzymes have similar catalytic domain (the Rossmann fold of the two enzymes superimpose with an rmsd = 3.5 Å for 159 matched Ca positions). (*B*) Comparison of Phe-tRNA^Phe^ binding to EF-Tu and to *Rgry*-CDPS. *Left* view, *T. thermophilus* EF-Tu:GDPNP:Phe-tRNA^Phe^ complex (PDB ID Code 1TTT, Nissen et al. 1995). *Middle* view, the Phe-tRNA^Phe^ molecules of the EF-Tu:GDPNP:Phe-tRNA^Phe^ and *Rgry*-CDPS:Phe-tRNA^Phe^ complexes were superimposed. The view shows how EF-Tu and *Rgry*-CDPS interact with different parts of the Phe-tRNA^Phe^ molecule. (*C*) Hijacking of elongator tRNAs by CDPSs. The figure shows that aa-tRNAs are diverted from their canonical role in ribosomal protein synthesis to catalyze the formation of two peptide bonds leading to the production of cyclodipeptides.

The structural homology of the CDPSs with class 1 aaRSs has been noted very early, the closest representatives being TyrRS and TrpRS (Vetting et al. 2010; Bonnefond et al. 2011; Sauguet et al. 2011). TyrRS and TrpRS belong to class1c aaRS (Giegé and Springer 2012). In contrast to other class I aaRS members, these two enzymes recognize their cognate tRNAs via the major groove of their acceptor stem. However, the 3′ end CCA of the tRNA is bound at the catalytic site with a bent conformation, which is a typical binding mode of class I aaRSs (Rould et al. 1989; Yaremchuk et al. 2002; Kobayashi et al. 2003; Shen et al. 2006; Yang et al. 2006; Carter and Wills 2018). In order to compare the tRNA binding mode of TyrRS and TrpRS with that of XYP-CDPS, we superimposed the Rossmann folds of the enzymes (Fig. 7A; Supplemental Fig. S8). The tRNA binding mode is totally different for CDPS and for TyrRS or TrpRS. The tyrosine and tryptophane amino acid substrates are located in the same binding pocket as the phenylalanyl moiety of Phe-tRNA in CDPS (P1 pocket). The ATP ligand of the two aaRS is located opposite to the A76 base of Phe-tRNA^Phe^ in the XYP-CDPS:Phe-tRNA^Phe^ complex. Interestingly, structural alignment of the two aaRS with the XYP-CDPS shows that the two enzymes are more divergent in the first part of their Rossmann folds (Fig. 7A; Supplemental Fig. S8). One may imagine that structural variability of this part of the Rossmann fold is linked to tRNA binding specificity. In human TrpRS-tRNA^Trp^, the 3′ end of tRNA^Trp^ bound at the catalytic site with a bent conformation is well defined in the structure (Shen et al. 2006). The tRNA^Trp^ acceptor arm is primarily recognized by an α-helix of the amino-terminal domain and has no base-speciﬁc interaction with the catalytic domain. It has been proposed that interaction of the A76 phosphate with the amino terminus of an α-helix is a constant feature facilitating the bent conformation of tRNAs bound to class I aaRS (Hol et al. 1978; Carter and Wills 2018). In contrast to class I aaRS (Carter and Wills 2018), there is no amino terminus of an α-helix stabilizing the conformation of A76 in CDPS. Taking into account the low resolution of the structure, it is not possible to give an atomic description of the CCA arm interactions with the protein. Nevertheless, interactions of β2 and β7 with the major groove of the acceptor stem likely favor the bent conformation of the tRNA (Figs. 5B, 6C). In this view, positively charged residues in this region (Fig. 2C) would play an important role. Moreover, the structure shows a channel formed by the two catalytic loops CL1 and CL2 located from either side of the CCA arm that contributes to its orientation toward the catalytic center. Notably, in *Cglo*-CDPS, CL1 loop is extended by seven residues. This extension could bring more surface interaction and thereby favor complex stability. In the case of *Rgry*-CDPS, the carboxy-terminal α9 helix is packed onto CL1. This interaction could also contribute to stabilize the bent conformation of the CCA arm.

In cells, CDPSs and other peptide bond-forming enzymes utilizing aa-tRNAs as substrates, such as L/F transferases or Fem aa-transferases (Fonvielle et al. 2013), are in competition with EF-Tu for the availability of aa-tRNA substrates. Consistent with this idea, the initial rate of L/F-transferase activity was shown to be reduced in the presence of an excess amount of EF-Tu (Suto et al., 2006). In order to better understand the molecular basis of such a competition, we compared the tRNA binding mode of EF-Tu with that of CDPSs. As shown in Figure 7B, aa-tRNAs are recognized through opposite sides by EF-Tu and CDPSs. Different types of bending of the acceptor ends enable the esterified amino acid to reach its binding pocket on the two proteins. We speculate that this may help CDPSs to hijack an aa-tRNA already bound to EF-Tu (Fig. 7C). Transient flipping of the aminoacylated CCA end toward the CDPS active site may lead to irreversible transfer of the amino acid to the catalytic serine. Alternatively, transient binding of CDPS to an aa-tRNA already bound to EF-Tu may accelerate its dissociation immediately followed by CDPS binding and catalysis. Interestingly, such an idea has already been proposed for two other enzymes interacting with aminoacylated-tRNA, the methionyl-tRNA_f_^Met^ transformylase (Schmitt et al. 1998) and the L/F-tRNA protein transferase (Suto et al. 2006). It is tempting to imagine that these opposite recognition modes generally contribute to the regulation of cellular processes.

## MATERIALS AND METHODS

### Bacterial strains and plasmids

*E. coli* XL1-blue and BL21 Rosetta pLacI-Rare (Invitrogen) have been used for cloning and expression experiments, respectively. The plasmid encoding *Cglo*-CDPS (pIJ196-CDPS23) was described (Jacques et al. 2015). It derives from pQE60 (Qiagen, ColE1 origin and ampicillin resistance) and carries the CDPS gene under the control of an IPTG-inducible PT5 promoter (Jacques et al. 2015). pIJ196-CDPS23 allows expression of the full-length CDPS with the addition of an A residue after the amino-terminal methionine and of the RS sequence followed by a His_6_ tag after the last residue.

### Site-directed mutagenesis

Thermo Scientific Phusion DNA polymerase was used to perform site-directed mutagenesis of *Cglo*-CDPS gene according to the manufacturer's protocol. Mutation of the catalytic serine into alanine was performed with the following oligonucleotides: 5′-GGCATTGCCATGCAGAGTCCGCATCAGATTAGC-3′ and 5′-GGACTCTGCATGGCAATGCCAATCACGATTTTAATC-3′. Deletions of amino- and carboxy-terminal parts of *Cglo*-CDPS residues were performed using the following oligonucleotides: Δ [1–9] 5′-GAGGAGAAATTAACCATGCTGGAACAGTTCATTCTGG-3′ and 5′-CCAGAATGAACTGTTCCAGCATGGTTAATTTCTCCTC-3′, Δ [251–293] 5′-CCGCTGCCGTTTATTGAACATCACCATCACCATCACTAAGC-3′ and 5′-GCTTAGTGATGGTGATGGTGATGTTCAATAAACGGCAGCGG-3′

### Production and purification of *Cglo*-CDPS variants

BL21 Rosetta pLacI-Rare cells were transformed with pIJ196-CDPS23 and grown in 2xTY medium containing 50 µg/mL ampicillin and 34 µg/mL chloramphenicol at 37°C until OD_600nm_ = 1. Protein expression was then induced by addition of isopropyl-β-D-thiogalactopyranoside at a final concentration of 0.5 mM at 20°C during 18 h. Cells were resuspended in lysis buffer (10 mM HEPES pH = 7.5, 500 mM NaCl, 3 mM β-mercaptoethanol) and disintegrated by sonication. *Cglo*-CDPS was purified via Talon Metal affinity chromatography (Clontech) using standard protocols followed by anion exchange chromatography (Q sepharose Fast Flow, GE Healthcare). Enzyme concentration was calculated using a computed extinction coefficient of 33140 M^−1^cm^−1^. About 1 mg of *Cglo*-CDPS was obtained from 1 L of culture.

### Production of CDPSs and characterization of cyclodipeptide-synthesizing activities

Recombinant CDPSs, wild-type *Cglo*-CDPS and variants *Cglo*-CDPS-S32A and *Cglo*-CDPS−[10-250] were expressed in *E. coli* BL21AI [pREP4] cells, respectively transformed by the plasmid pIJ196-CDPS23 (Jacques et al. 2015), and the corresponding mutated plasmids (see above). The strains were grown in 10 mL M9 minimum medium supplemented with trace elements and vitamins (Gondry et al. 2009), 0.5% glucose, 200 µg/mL ampicillin and 25 µg/mL kanamycin. After overnight incubation at 37°C, this starter culture was used to inoculate 200 mL of the same medium except that glucose was replaced by 0.5% glycerol. Bacteria were grown at 37°C until the OD_600_ reached 0.6. The expression of CDPSs was induced by the addition of IPTG (2 mM final concentration) and cultivation was continued for 18 h at 20°C. The cultures were then centrifugated at 4000*g* for 45 min. Supernatants were analyzed for the presence of cyclodipeptides and CDPS expression was analyzed in cell pellets, as follows.

Supernatants were acidified with trifluoroacetic acid at 2% v:v final, and stored at −20°C. They were then analyzed by LC-MS/MS analyses on an Elute SP HPLC chain (Bruker Daltonik GmbH) coupled via a split system to an amazon SL ion trap mass spectrometer (Bruker Daltonik GmbH) set in positive mode. Samples were loaded onto a C18-PFP column (4.6 mm × 150 mm, 3 µm, 100 Å, ACE) developed over 20 min with the linear gradient 0%–50% (v/v) (solvent A: 0.1% [v/v] formic acid in H2O, solvent B: 0.1% [v/v] formic acid in acetonitrile/H2O [90/10], flow rate, 0.6 mL/min). Cyclodipeptides were detected and identified as previously described (Fig. 2A,B; Canu et al. 2019).

Cell pellets were frozen at −80°C and treated as described except that they were suspended in only 4 mL of ice-cold lysis buffer (100 mM Tris HCl, pH 8.0, 150 mM NaCl, 5% glycerol [v/v], 10 mM beta-mercaptoethanol, 1 mM phenylmethylsulfonylfluoride) (Gondry et al. 2009). An amount of 550 µL of the soluble fractions were subjected to spin purification using Micro Bio-Spin chromatography columns filled with 100 µL of Protino Ni-NTA Agarose resin. The columns were washed with 100 µL of buffer A containing 80 mM imidazole and the tagged proteins were eluted with 100 µL buffer A containing 350 mM imidazole. Eluted fractions were analyzed by 12% SDS-PAGE with Coomassie blue staining (Supplemental Fig. S7A).

CD spectra of *Cglo-*CDPS-S32A and *Cglo-*CDPS-[10-250] were recorded using a Jobin-Yvon Mark VI circular dichrograph at a scan speed of 0.2 nm/sec. The protein concentrations were 5 µM. Quartz cuvettes with 0.1 cm path length per compartment were used. The results are presented as normalized Δε values on the basis of the amino acid mean residue mass of 110 Da (Supplemental Fig. S7C).

### Production and aminoacylation of tRNA^Phe^

A synthetic gene for *E. coli* tRNA^Phe^ was assembled from six overlapping oligonucleotides and cloned into pBST-NAV2 (Meinnel et al. 1992). *E. coli* tRNA^Phe^ was then overexpressed in JM101Tr cells transformed with pBST-NAV2-tRNA^Phe^ in a 1 L 24 h culture at 37°C. tRNA^Phe^ was purified as described previously for tRNA_f_^Met^ (Mechulam et al. 2007). Briefly, a whole tRNA extract was prepared according to the Zubay procedure (Zubay 1962). The overexpressed tRNA^Phe^ was then purified by an anion exchange step on a Q-Hiload column (16 × 100 mm) equilibrated in 20 mM Tris-HCl pH 7.6, 200 mM NaCl, 8 mM MgCl_2_, 0.1 mM EDTA. tRNAs were eluted using a 350 mM to 550 mM NaCl gradient in the same buffer. About 10 mg of tRNA^Phe^ was obtained from 1 L of *E. coli* culture. ^14^C Phenylalanine acceptance of the tRNA^Phe^ preparations ranged between 1450 and 1600 pmol/A_260_ unit. tRNA (10 µM) was aminoacylated during 20 min at 25°C using purified *E. coli* PheRS (1 µM) in a buffer containing 10 mM HEPES pH 7.5, 150 mM NaCl, 0.1 mM EDTA, 8 mM MgCl_2_, 2 mM ATP and 400 µM Phenylalanine. PheRS was overproduced in *E. coli* IBPC1671 from plasmid pBH16D (Mechulam et al. 1987) and purified using standard chromatographic procedures. After aminoacylation, tRNA was ethanol precipitated in the presence of 166 mM sodium acetate pH 5.0. The aminoacylated tRNA was then purified onto a MonoQ column (GE-Healthcare) using a 0 mM to 400 mM NaCl gradient followed by a 800 mM NaCl eluting step. The recovered fractions were ethanol precipitated and the aminoacylated tRNA was finally resuspended in buffer A containing 10 mM MOPS pH 6.7, 200 mM NaCl, 8 mM MgCl_2_, and 5 mM 2-mercaptoethanol.

### Gel filtration analysis of CDPS:tRNA mixtures

1.2 molar excess of aminoacylated or nonaminoacylated tRNA^Phe^ were mixed with *Cglo*-CDPS-S32A variant in buffer A. The mixtures were kept 15 min at 4°C before analysis by size exclusion chromatography onto a Superdex 200 Increase 10/300 column (0.75 mL/min) equilibrated in buffer A. After gel filtration, fractions were analyzed by SDS-PAGE and gels were successively stained with BET and Coomassie blue to reveal tRNAs and proteins, respectively (Fig. 3; Supplemental Fig. S2).

### Electrophoresis mobility shift assay

Electrophoretic analyses were performed on native 12% acrylamide gels in buffer A containing 90 mM Tris pH 8.3, 90 mM sodium borate, 2 mM EDTA and 0.5 mM MgCl_2_. tRNA (75 nM) was incubated with increasing concentrations of *Cglo*-CDPS (from 0.1 to 15 µM) for 20 min at 4°C in buffer A. Before loading, 10 µL of the mixture was supplemented with 2 µL loading buffer (0.0025% formamide blue, 0.0025% xylene cyanol, 40% glycerol). The gels were run at 100 V for 2 h at 4 °C and then stained successively with SYBR gold (Thermofisher) to reveal the bands containing tRNA and Coomassie blue to reveal the bands containing protein.

### Crystallization and structure determination of *E. coli* tRNA^Phe^ and of *Cglo*-CDPS-S32A:Phe-tRNA^Phe^ complex

Freshly gel filtration purified CDPS-S32A:Phe-tRNA^Phe^ complex, as described in Figure 3, was used in vapor diffusion crystallization trials at 4°C using hanging drops obtained by mixing 2 µL of *Cglo*-CDPS-S32A:Phe-tRNA^Phe^ complex (150 µM) with 1.6 µL of precipitating solution (Table 1). Two types of crystal were obtained. Before data collection, both crystal types were soaked in a cryoprotecting solution containing the precipitating solution plus 30% (v/v) glycerol. Diffraction data were collected at 100 K at the Proxima 2 beamline (SOLEIL Synchrotron). Diffraction images were analyzed with XDS (Kabsch 1988) and further processed with programs of the CCP4 package (Collaborative Computational Project Number 4, 1994). Both crystal types belonged to a hexagonal space group (Table 1). Crystals containing only tRNA^Phe^ diffracted to 3.1 Å resolution and those containing the CDPS-S32A:Phe-tRNA^Phe^ complex diffracted to 5.0 Å resolution. In a first step, the structure of *E. coli* tRNA^Phe^ was solved by molecular replacement with PHASER (McCoy et al. 2007) using the [1–71] region of *E. coli* tRNA^Phe^ from 4YCO PDB entry (Byrne et al. 2015) as a search model (*Z*-score = 18.1) and refined with PHENIX (Adams et al. 2010). The final structure of *E. coli* tRNA^Phe^ contains nucleotides 1 to 75.

A highly redundant data set was collected from one crystal containing *Cglo-*CDPS-S32A:Phe-tRNA^Phe^ complex to 5 Å resolution using the helical collection mode at Proxima 2 (Table 1). After data processing with XDS (Kabsch 1988), the data set was corrected for anisotropy using the STARANISO server (http://staraniso.globalphasing.org/cgi-bin/staraniso.cgi, Table 1) (Vonrhein et al. 2018). The structure of *Cglo*-CDPS-S32A:Phe-tRNA^Phe^ complex was solved by molecular replacement with PHASER (Storoni et al. 2004) using two ensembles. The first one corresponded to the core domain of *Rgry*-CDPS (residues 12–223, PDB:5MLP [Bourgeois et al. 2018]) and the second one to the presently determined 2–71 segment of the structure of *E. coli* tRNA^Phe^. For each ensemble, a solution with a high *Z*-score value was found (*Z* = 12.3 and *Z* = 6.8). The molecular replacement 2mF_o_ − DF_c_ electron density map was of good quality. In particular, additional electron density was visible for nucleotides 72–75 of the tRNA that had been omitted in the molecular replacement model. The secondary structure elements of *Cglo*-CDPS were placed in the electron density. Considering the low diffraction limit, the protein model was built as a polyalanine model with only a few side chains to avoid data overfitting. Coordinates were refined through several cycles of manual adjustment with Coot (Emsley et al. 2010) and positional refinement with PHENIX (Adams et al. 2010) with restraints on secondary structure elements of the protein and of the tRNA. In the last steps of refinement, we modeled the aminoacylated A76 adenosine as described in the Results section. The final model refined to 5 Å resolution contained all nucleotides of Phe-tRNA^Phe^ and residues 9–69 and 75–268 of *Cglo*-CDPS (Table 1).

### SAXS data collection

SAXS experiments were conducted at the SWING beamline of the SOLEIL Synchrotron (λ = 1.033 Å). Data were collected in the Q-range 0.008–0.6 Å^−1^ (Q = 4πsinθ/λ, 2θ is the scattering angle). All solutions were circulated in a thermostated (15°C) quartz capillary with a diameter of 1.5 mm and a wall thickness of 10 µm, positioned within a vacuum chamber. For data collection, purified *Cglo*-CDPS-S32A:Phe-tRNA^Phe^ complex (c.a 20 nanomoles in 100–200 µL) was injected onto a size-exclusion Superdex 200 increase 3.2/300 column (GE Healthcare) using an Agilent High Performance Liquid Chromatography system and eluted directly in buffer A into the SAXS capillary cell at a flow rate of 0.075 mL/min. SAXS data were collected online, with a frame duration of 1.0 sec. A large number of frames were collected during the first minutes of the elution and averaged to account for buffer scattering, which was subsequently subtracted from the signal during elution of the complex. Selected curves corresponding to the main elution peak were averaged on the basis of identical shapes (David and Perez 2009). Data reduction to absolute units, frame averaging and subtraction, were performed using FOXTROT (http://www.synchrotron-soleil.fr/Recherche/LignesLumiere/SWING). All subsequent data processing, analysis and modeling steps were carried out with PRIMUS and other programs of the ATSAS suite (Konarev et al. 2006). Scattered intensity curves were calculated from the atomic coordinates of the crystallographic structures using CRYSOL (Svergun et al. 1995) with 50 harmonics. This program was also used to fit the calculated curve to the experimental one, by adjusting the excluded volume, the averaged atomic radius and the contrast of the hydration layer surrounding the particle in solution.

## DATA DEPOSITION

Coordinates and structure factors have been deposited in the PDB with accession numbers 6Y3G for *E. coli* overproduced tRNA^Phe^ and 6Y4B for *Cglo*-CDPS-S32A:Phe-tRNA^Phe^ complex. SAXS data have been deposited at the SASDB (accession code SASDJT4).

## SUPPLEMENTAL MATERIAL

Supplemental material is available for this article.

## Supplementary Material

Supplemental Material
