# Supplementary material for: Structural basis of the interaction between cyclodipeptide synthases and aminoacylated tRNA substrates
Source: RNA. 2020 Nov;26(11):1589–602. doi: 10.1261/rna.075184.120 (PMC7566563; doi:10.1261/rna.075184.120)
Supplement: Supplemental Material [file supp_26_11_1589__index.html]

Structural basis of the interaction between cyclodipeptide synthases and aminoacylated tRNA substrates — Supplemental Material 

# Structural basis of the interaction between cyclodipeptide synthases and aminoacylated tRNA substrates

## Supplemental Material

- Supplemental\_Figures\_S1-S8.pdf
