# Supplementary material for: Structural basis of the interaction between cyclodipeptide synthases and aminoacylated tRNA substrates
Source: RNA. 2020 Nov;26(11):1589–602. doi: 10.1261/rna.075184.120 (PMC7566563; doi:10.1261/rna.075184.120)
Supplement: Supplemental Material [file supp_075184.120_Supplemental_Figures_S1-S8.pdf]

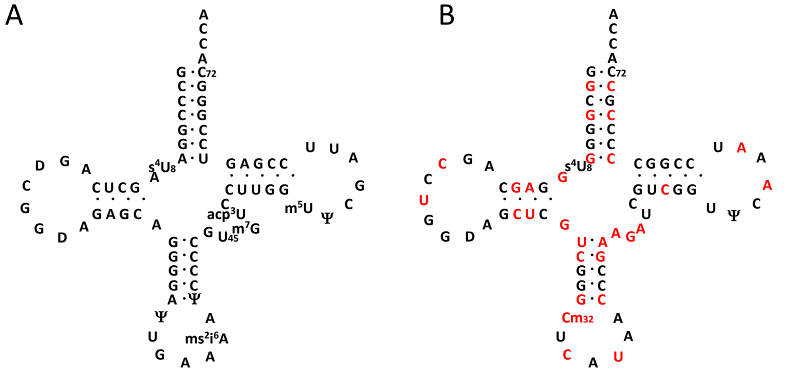

E. coli tRNA^{Phe} *E. coli* tRNA$_f^{Met}$G$_1$-C$_{72}$

Supplemental Figure S1: Cloverleaf representations of overproduced tRNAs.

A-*E. coli* tRNA^{Phe}. B-*E. coli* tRNA$_f^{Met}$G1-C72. Post-transcriptional modifications of mature tRNAs are indicated.

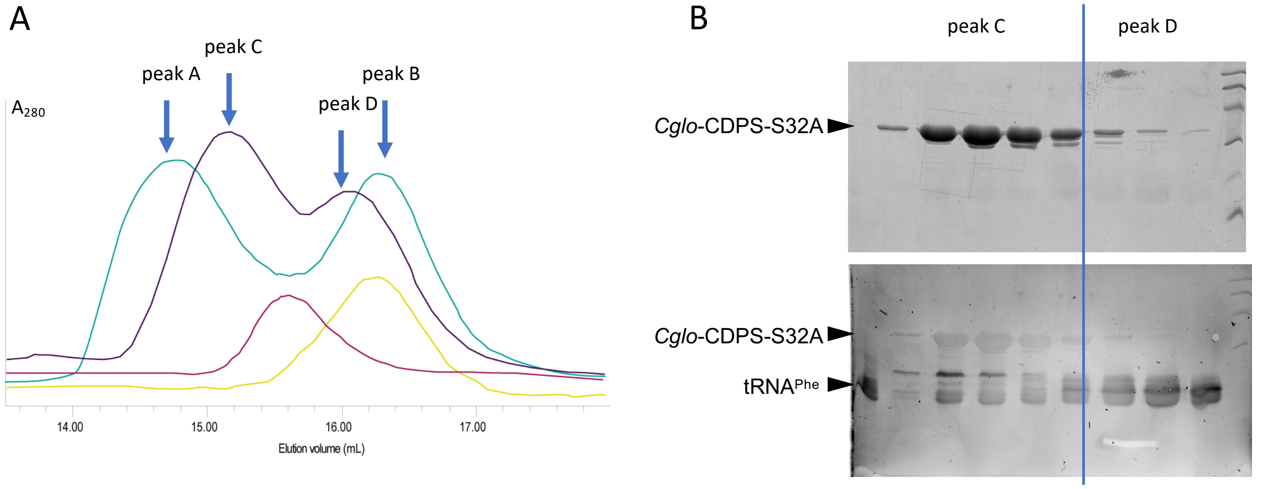

Supplemental Figure S2: Gel filtration analysis of *Cglo*-CDPS:tRNA complex.
A-Gel filtration chromatograms for *Cglo*-CDPS-S32A (pink), Phe-tRNA^{Phe} (yellow), *Cglo*-CDPS-S32A:Phe-tRNA^{Phe} (green) and *Cglo*-CDPS-S32A:tRNA^{Phe} (purple).
B-SDS-PAGE analysis of peaks C and D. Gels were stained using ethidium bromide (lower part) and then with Coomassie blue (upper part). The molecular weight marker (GE-Healthcare) is shown to the right part of the gel. Visible bands correspond to protein standards with molecular weight of 97, 66, 45, 30, 20, 14.4 kDa from top to bottom. SDS-PAGE analysis of peaks A and B is shown in Figure 2B.

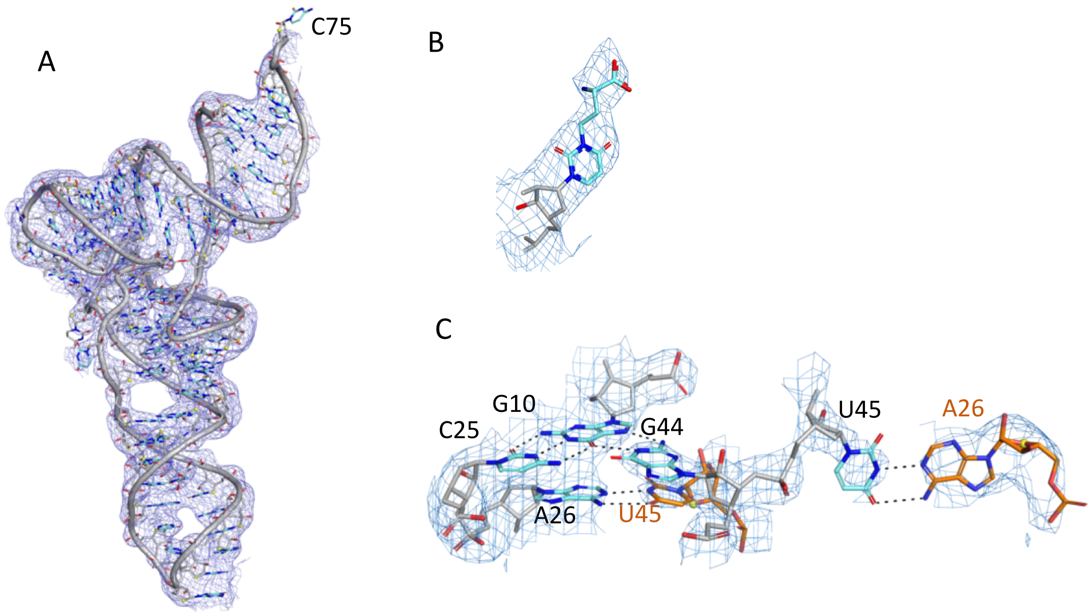

Supplemental Figure S3: Crystal structure of overproduced *E. coli* tRNA^{Phe}

A-Global view of *E. coli* overproduced tRNA^{Phe} and its electron density contoured at 1 σ. B-Closeup of acp^{3}U47. The 2mFo-DFc electron density map is contoured at 0.6 σ. C-Closeup of the triplet base pair. C25:G10:G44 and its packing environment. A26 is unpaired and U45 is flipped out. The 2mFo-DFc electron density map is contoured at 1.8 s. Residues belonging to a neighbor molecule are colored in orange whereas those of the reference molecule are colored in grey and cyan sticks.

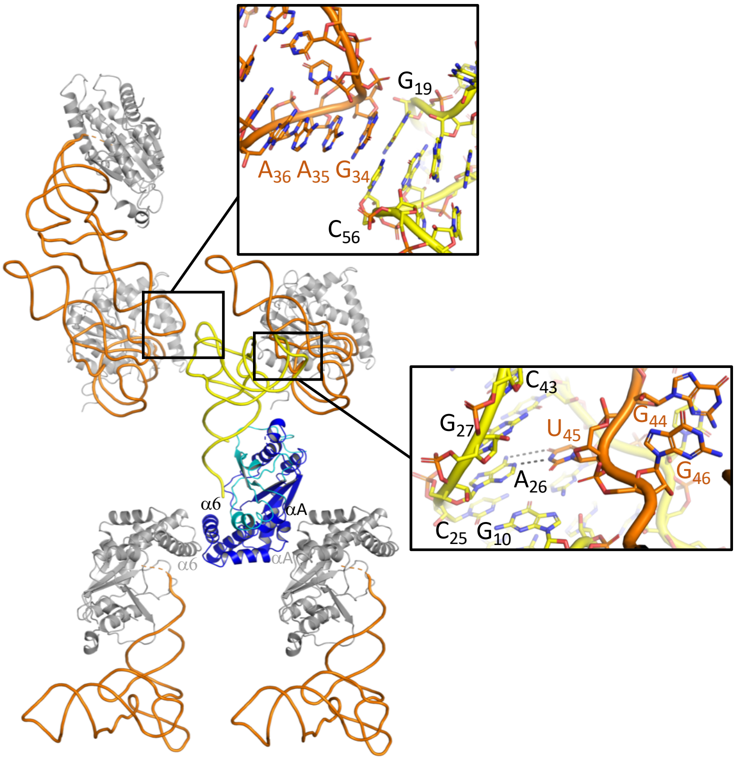

Supplemental Figure S4: Crystal packing interactions for the *Cglo*-CDPS-S32A:Phe-tRNA^{Phe} complex.
The central view shows arrangement of the *Cglo*-CDPS-S32A:Phe-tRNA^{Phe} molecules within the P6$_1$22 crystals. Two contact points are boxed and detailed in the closeups. The reference molecule of the asymmetric unit is in blue (*Cglo*-CDPS) and yellow (tRNA) and the neighboring molecules are in grey (*Cglo*-CDPS) and orange (tRNA).

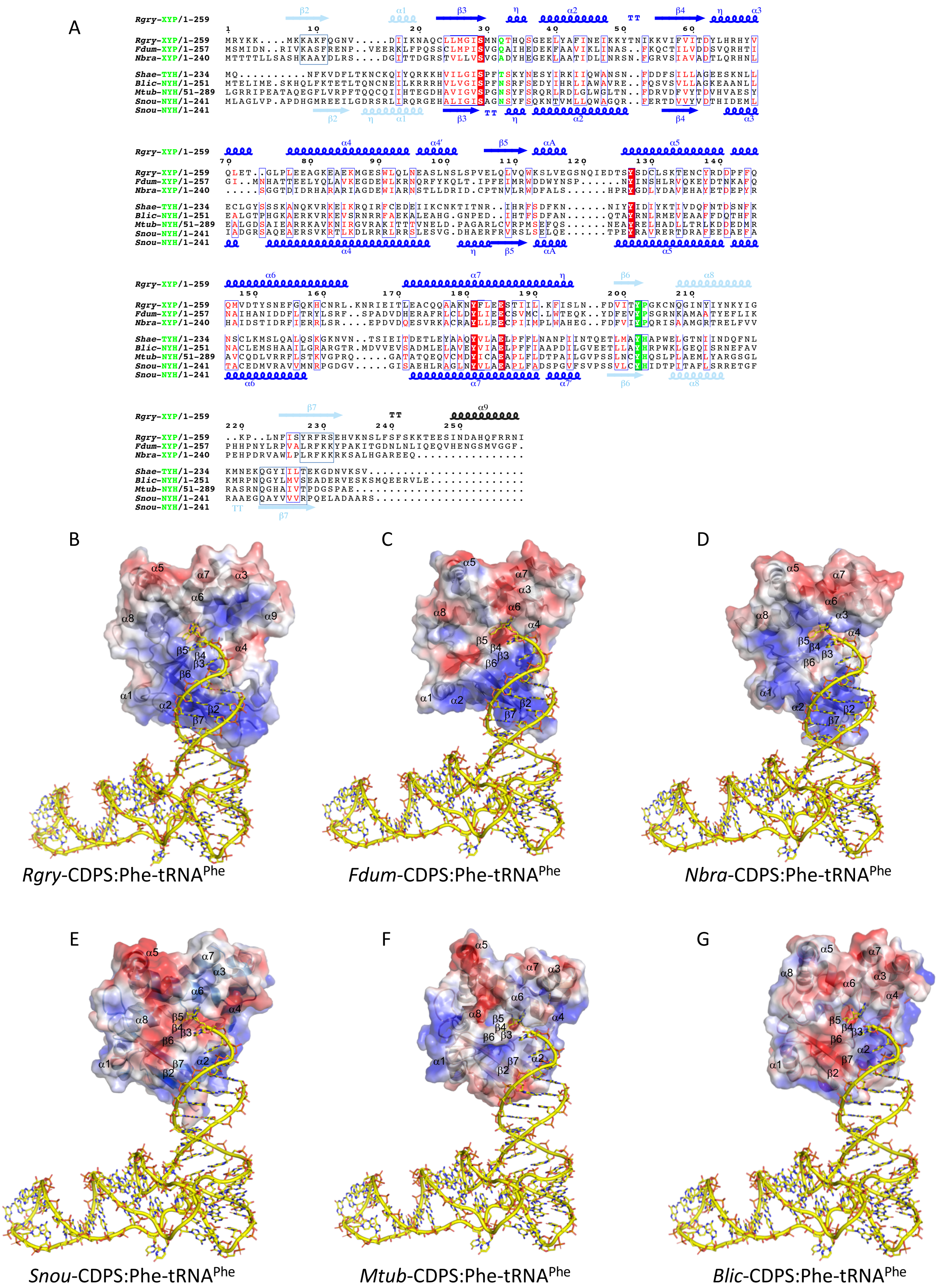

Supplemental Figure S5: XYP-CDPS:tRNA docking models.

A-Sequence alignment of NYH and XYP CDPSs. The figure was drawn with Espript (Gouet et al., 1999). When the percentage of identity is higher that 70% in the seven sequences aligned, residues are colored in red and framed in blue. In case of strict identity, residues are in white on a red background. Secondary structures of *Rgry*-CDPS are indicated at the top of the alignment and those of *Snou*-CDPS are indicated at the bottom. The first half of the Rossmann fold is colored in cyan and the second half of the Rossmann fold is colored in blue. The NYH and XYP motifs are colored in green. Conserved regions in β2 and β7 are boxed. "Reprinted with modifications from Bourgeois et al., 2018, with permission (Licence number 4775930882792)". B, C, D-The docking models are deduced from superimposition of the corresponding XYP-CDPS onto *Rgry*-CDPS:Phe-tRNA^{Phe} complex. The protein electrostatic potential maps are represented (blue, positive; red, negative; white, neutral with the same scale from -2.8 to +2.8 k_BT/e_c) and the tRNA is shown in sticks and cartoon. The views emphasize the binding of the acceptor arm of the tRNA by the positively charged protein area containing β2-β7 (see also Figure 4). The name of the protein:tRNA complex is indicated below the structure. *Rgry*, *Rickettsiella grylli*; *Nbra*, *Nocardia brasiliensis*; *Fdum*, *Fluoribacter dumoffii*. E, F, G-same as B, C, D but for the NYH-CDPSs. Comparison of views B, C, D with the views E, F, G highlights that NYH-CDPSs do not possess the positively charged area containing β2-β7. *Snou*, *Streptomyces noursei*; *Mtub*, *Mycobacterium tuberculosis* (formerly called Rv2275); *Blic, Bacillus licheniformis* (formerly called YvmC).

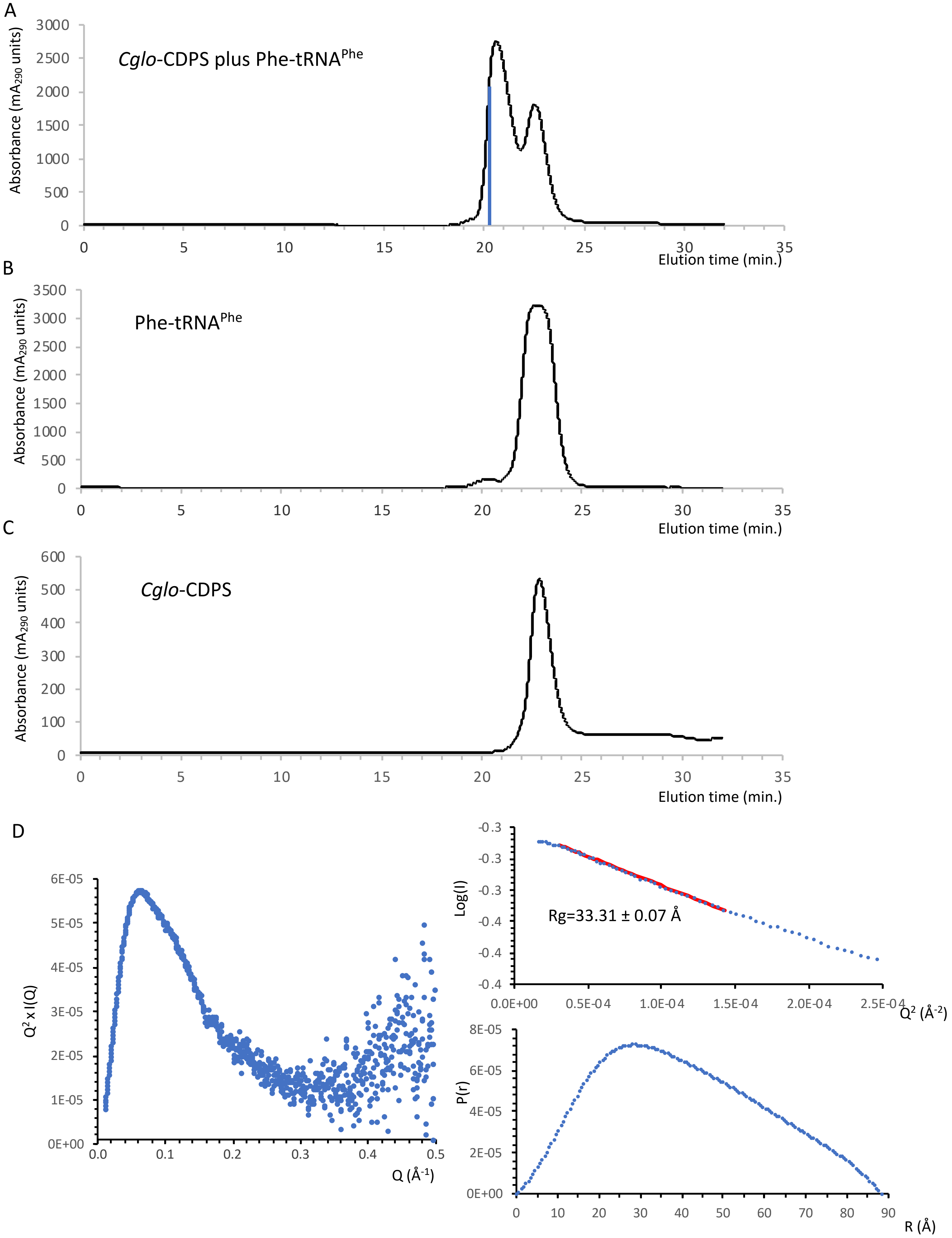

Supplemental Figure S6: Small-angle X-ray scattering analysis of the *Cglo*-CDPS:Phe-tRNAPhe complex. Panel A shows the size exclusion chromatogram in the SAXS experiment corresponding to the mixture of *Cglo*-CDPS and Phe-$tRNA^{Phe}$. Chromatograms corresponding to Phe-$tRNA^{Phe}$ (B) and *Cglo*-CDPS (C) are shown for comparison. The blue bar in panel A shows the positions of the scattering frames averaged for the SAXS curve (Fig. 5D). This region was chosen to minimize the contribution of excess tRNA while maintaining sufficient scattering signal from the eluted complex. Panel D: left, Kratky plot of the scattering data of the complex (Fig. 5D) with a bell shape typical of a folded macromolecule; right top, Guinier plot; right bottom: distance distribution with an Rmax of 88 Å.

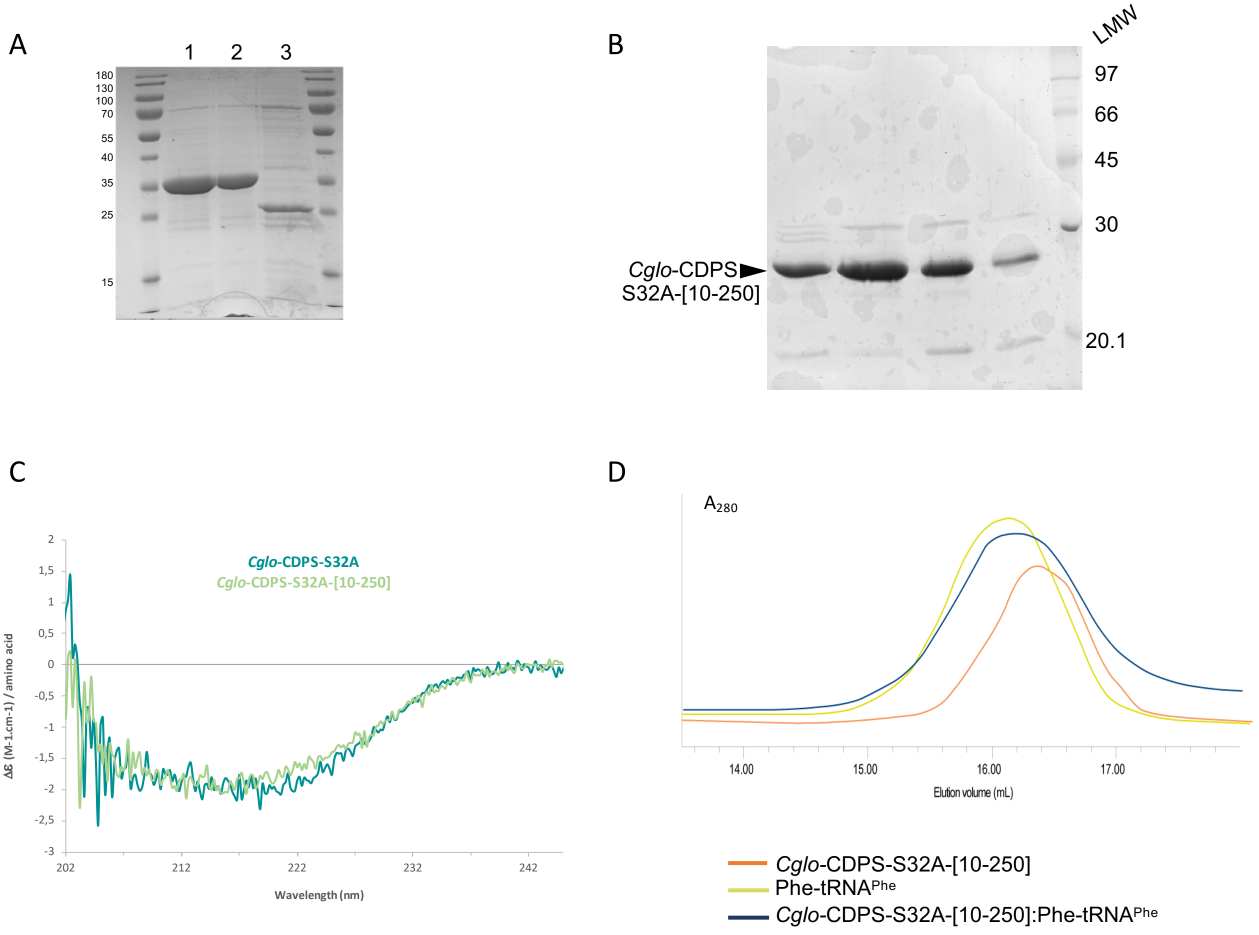

Supplemental Figure S7: Characterization of *Cglo*-CDPS-[10-250] variant.
A-*Cglo*-CDPSs (wild-type, variants S32A and [10-250]) were expressed in *E. coli* BL21AI and were grown as described in Materials and Methods. Overexpression of each soluble protein was evaluated, after micropurification on Ni-NTA resin, by SDS-PAGE analysis and the production of cyclodipeptide was measured as described in the Materials and Methods section. 1- *Cglo*-CDPS; 2- *Cglo*-CDPS-S32A; 3- *Cglo*-CDPS-[10-250]. B-SDS-PAGE analysis showing purification of *Cglo*-CDPS-S32A-[10-250] variant on Superdex 200 HR (GE Healthcare). C- CD spectra of *Cglo*-CDPS-S32A and *Cglo*-CDPS-[10-250]. D-Gel filtration chromatograms for *Cglo*-CDPS-S32A-[10-250] (orange line), Phe-$tRNA^{Phe}$ (yellow line), *Cglo*-CDPS-S32-[10-250]: Phe-$tRNA^{Phe}$ (blue line).

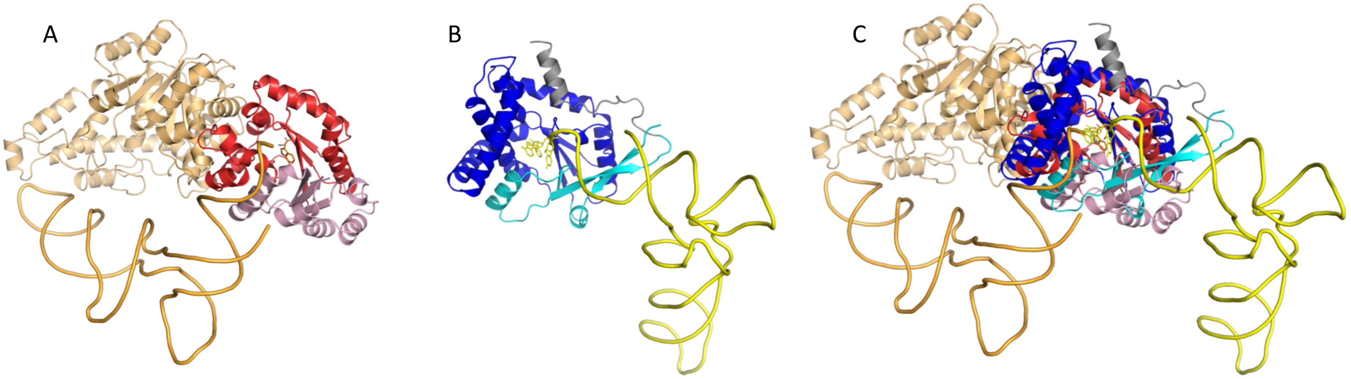

Supplemental Figure S8: Comparison of CDPS and hTrpRS tRNA binding modes.

Structural alignment of *Rgry*-CDPS:Phe-tRNA^{Phe} with *human* TrpRS:tRNA^{Trp} (PDB ID Code 2DR2, Shen et al., 2006).

A-*human* TrpRS:tRNA^{Trp} complex is represented in cartoon. One monomer is in light orange and the N-terminal domain of the second monomer is in colored in pink (first part of the Rossmann fold) and in dark red (second part of the Rossmann fold). Tryptophane is shown in sticks.

B-*Rgry*-CDPS:Phe-tRNA^{Phe} model. Phe-A76 and C75 are shown in yellow sticks. The color code is the same as in Figure 5.

C-Superimposition of the two structures. The view shows that the tRNA binding mode is different for *Rgry*-CDPS and for TrpRS even if the two enzymes have similar catalytic domain (the Rossmann fold of the two enzymes superimpose with an rmsd=3.55 Å for 152 matched Cα positions).
